# Association between neonatal conditions and anthropometric indicators among children attending daycare centers in Southern Brazil

**DOI:** 10.1590/1980-220X-REEUSP-2025-0598en

**Published:** 2026-07-24

**Authors:** Luiz Felipe de Paiva Lourenção, Lara Campos Borim, Laura Thaciane Rodrigues Cassiano, Roseli Oselka Saccardo Sarni, Rosangela Silva

**Affiliations:** 1 Universidade Federal de Alfenas, Faculdade de Nutrição, Alfenas, MG, Brazil.; 2 Universidade Federal de São Paulo, Escola Paulista de Medicina, São Paulo, SP, Brazil.; 3 Centro Universitário FMABC, Santo André, SP, Brazil.

**Keywords:** Child Nutrition, Growth and Development, Food Security, Anthropometry, Nutrição da Criança, Crescimento e Desenvolvimento, Segurança Alimentar, Antropometria

## Abstract

**Objective:**

To investigate the association between neonatal conditions and anthropometric indicators and to describe food insecurity among children attending daycare centers.

**Methods:**

Cross-sectional study with 139 children enrolled in Early Childhood Education Centers in southern Minas Gerais, Brazil. Data on neonatal conditions, socioeconomic characteristics, food insecurity, and nutritional status were collected. Height-for-age (HAZ) and body mass index-for-age (BAZ) were classified according to the World Health Organization standards. Poisson regression with robust variance was used to estimate prevalence ratios (PR).

**Results:**

The prevalence of overweight was 34.4%, and short stature was observed in 22.6% of children. Overweight prevalence was 34.4% and short stature 22.6%. BAZ was associated with birth weight (PR = 1.0016; 95%CI:1.0004–1.0028), food insecurity, socioeconomic status, and high-risk pregnancy. Higher food insecurity levels showed higher PRs for BAZ, while lower socioeconomic classes were inversely associated. No associations were observed for HAZ.

**Conclusion:**

BAZ was associated with perinatal and socioeconomic factors, highlighting early-life determinants of excess weight and the influence of food insecurity, reinforcing the need for early interventions.

## RESUMO

**Objetivo:**

Investigar a associação entre condições neonatais e indicadores antropométricos, bem como descrever a insegurança alimentar entre crianças frequentadoras de creches.

**Métodos:**

Estudo transversal com 139 crianças matriculadas em Centros de Educação Infantil no sul de Minas Gerais, Brasil. Foram coletados dados sobre condições neonatais, características socioeconômicas, insegurança alimentar e estado nutricional. Os índices estatura para idade (E/I) e índice de massa corporal para idade (IMC/I) foram classificados de acordo com os padrões da Organização Mundial da Saúde. Utilizou-se regressão de Poisson com variância robusta para estimar razões de prevalência (RP).

**Resultados:**

A prevalência de excesso de peso foi de 34,4%, e baixa estatura foi observada em 22,6% das crianças. O IMC/I esteve associado ao peso ao nascer (RP = 1,0016; IC95%: 1,0004–1,0028), insegurança alimentar, nível socioeconômico e gestação de alto risco. Níveis mais elevados de insegurança alimentar apresentaram maiores razões de prevalência para IMC/I, enquanto classes socioeconômicas mais baixas mostraram associação inversa. Não foram observadas associações para E/I.

**Conclusão:**

O IMC/I esteve associado a fatores perinatais e socioeconômicos, destacando determinantes precoces do excesso de peso e a influência da insegurança alimentar, reforçando a necessidade de intervenções precoces.

## INTRODUCTION

Adequate pregnancy and birth conditions, exclusive breastfeeding up to six months of age, and healthy complementary feeding play an essential role throughout the life course, particularly in early childhood, contributing to adequate growth and development, the formation of healthy habits, and health promotion^(1,2)^.

During the first two years of life, food choices require special attention, as this period is critical for the establishment of eating habits. Diets based on fresh or minimally processed foods should form the foundation of both the child’s and the family’s diet. In this context, the diversity and the manner in which foods are offered influence the development of taste preferences, children’s relationship with food, and their nutritional status. Health care and nutrition strategies implemented during this period may have repercussions throughout the life course^(3)^. Therefore, children’s nutritional status is considered a sensitive indicator of health and quality of life, reflecting the development model of a given society^(4)^.

Recent studies indicate that children’s diets are characterized by low dietary diversity, reduced consumption of fresh or minimally processed foods, and early exposure to ultraprocessed foods, particularly in low-income and low-education households. This dietary pattern is associated with an increased risk of overweight, obesity, and noncommunicable diseases (NCDs) later in life^(5,6,7)^. Ultra-processed foods are industrial formulations with poor nutritional quality, characterized by high energy density, elevated levels of fats, sugars, and sodium, and low fiber content, resulting from multiple industrial processing steps and the addition of substances to enhance palatability and shelf life^(8)^.

Birth conditions play a critical role in the development of nutritional disorders, including undernutrition and excess weight. Birth weight is an important indicator of neonatal nutritional status, and low birth weight (LBW) is recognized as a risk factor for infant and post-neonatal morbidity and mortality, as well as for delayed child development^(9,10)^.

A diet rich in ultra-processed foods during early childhood negatively affects growth and development and contributes to excess weight in this population. This dietary pattern has been linked to long-term health consequences, including obesity, diabetes, hypertension, and dyslipidemia^(11,12)^. Although previous studies have explored the role of early-life conditions and dietary patterns in child growth, evidence on the association between neonatal conditions and anthropometric indicators in children attending daycare centers, particularly in small Brazilian municipalities, remains limited. Thus, this study aimed to investigate the relationship between neonatal conditions and anthropometric indicators and to describe food and nutrition security among children enrolled in daycare centers.

## METHOD

### Type of Study

This was a cross-sectional study conducted among children enrolled in municipal Early Childhood Education Centers in a municipality in southern Minas Gerais, Brazil.

### Study Setting

The study was carried out in three municipal Early Childhood Education Centers (*CEI*s) located in the same municipality. Data collection took place between April and July 2022. The CEIs operate on a full-time basis and provide four daily meals (breakfast, lunch, afternoon snack, and dinner), designed to meet at least 70% of daily nutritional requirements for energy, macronutrients, and priority micronutrients (vitamins A and C, calcium, and iron).

### Participants and Sample

All children enrolled in the selected Early Childhood Education Centers during the data collection period were considered eligible for participation, characterizing a census approach rather than probabilistic sampling.

A total of 174 children met the eligibility criteria. Of these, 23 did not participate due to refusal by parents or legal guardians to provide informed consent, and 12 were excluded due to incomplete data in key study variables. Thus, the final analytical sample consisted of 139 children (79.8% of the eligible population). No significant differences in age and sex distribution were observed between participants and non-participants.

Participation was voluntary and dependent on parental authorization, which may have contributed to non-participation. Children with neurological impairments and/or those using corticosteroid medications were excluded from the study. Therefore, the final sample resulted from the eligible population during the study period and was not based on a prior sample size calculation.

### Variables

The primary outcomes of the study were indicators of nutritional status, assessed by Height-for-Age (HAZ) and Body Mass Index-for-Age (BAZ) Z-scores, classified according to the World Health Organization (WHO) reference standards (Z-scores < -2 indicating deficit and > +2 indicating excess weight).

Exposure variables included gestational age, birth weight, birth length, breastfeeding practices, and household food and nutrition security. Sociodemographic variables were also collected to characterize the study population.

### Data Collection Procedures

Data were collected using a structured and standardized questionnaire administered to the parents or legal guardians. The questionnaire included information on gestational history, neonatal and birth conditions, breastfeeding practices, and socioeconomic characteristics.

Socioeconomic status was assessed using the Brazilian Economic Classification Criteria established by the Brazilian Association of Research Companies (*ABE*P)^(13)^.

To minimize information bias, data collection was performed by trained researchers using standardized instruments and procedures.

### Assessment of Nutritional Status

Anthropometric measurements of weight and height were obtained at the Early Childhood Education Centers following standardized procedures recommended by the World Health Organization (WHO). Weight and height measurements were used to calculate HAZ and BAZ, classified according to WHO reference standards. Data processing and classification were performed using WHO Anthro ^®^ software^(14)^.

### Food and Nutrition Security Assessment

Food and nutrition security was assessed using the Brazilian Food Insecurity Scale (*EBIA*), a validated psychometric instrument composed of 14 dichotomous questions (“yes” or “no”) that evaluate household food access based on the perception and experience of food insecurity over the previous three months^(15)^.

### Statistical Analysis

Descriptive analyses were performed to characterize the study population, using absolute and relative frequencies for categorical variables, along with their respective 95% confidence intervals (95% CI), calculated assuming a binomial distribution. Continuous variables were described using means and standard deviations, according to their distribution.

Bivariate analyses were conducted to explore relationships between exposure variables and the outcomes. Pearson’s chisquare test was applied when assumptions were met, and Fisher’s exact test was used in cases of small expected cell frequencies to ensure the validity of statistical inference.

Multivariable analyses were performed using generalized linear models to estimate prevalence ratios (PR) and corresponding 95% confidence intervals. Considering the cross-sectional design and the non-rare nature of the outcomes, Poisson regression with robust variance was adopted as the primary modeling strategy.

For the BAZ outcome, a Poisson model with robust variance was used, assuming an approximately equidispersed distribution. For the HAZ outcome, a quasi-Poisson model was applied to account for dispersion parameters differing from unity, improving the adequacy of variance estimation.

Variables with p < 0.20 in bivariate analyses were included in the multivariable models, and statistical significance was defined as p < 0.05. Model estimates were interpreted with caution when confidence intervals were wide or included the null value, indicating limited precision.

All statistical analyses were performed using R software, version 4.5.2.

### Ethical Aspects

The study was approved by the Research Ethics Committee of the Universidade Federal de São Paulo (UNIFESP), under opinion number 3.936.371, dated 2020. All procedures were conducted in accordance with the ethical guidelines and regulatory standards for research involving human beings, as established by the Brazilian National Health Council Resolutions No. 466/2012 and Nº. 510/2016. Parents or legal guardians received detailed information about the study objectives and procedures and provided written informed consent prior to the participation of the children.

## RESULTS

A total of 139 children were included; most were male (56.8%; 95% CI: 48.2–65.2) and aged 24–35 months (38.9%; 95% CI: 30.7–47.5). Most children were classified as White (68.2%), and the predominant socioeconomic class was B2 (34.3%). Food and nutrition insecurity was observed in 47.5% of households, predominantly at the mild level (35.8%), while moderate and severe forms were less frequent (8.8% and 2.9%, respectively). Food security was observed in 52.6% of households.

Most mothers had finished high school (48.1%), and 33.8% had higher education. Preterm birth occurred in 10.2% of cases, and 11.0% of pregnancies were classified as high-risk. Gestational complications were reported in 24.6% of cases. Nearly all deliveries occurred in hospital settings (98.6%), with cesarean section as the predominant mode (62.6%). Postpartum complications were reported in 9.9% of cases. Most children were born with adequate birth weight (71.4%), while low birth weight and macrosomia were less frequent (3.8% and 5.3%, respectively). Adequate birth length and normal head circumference were observed in 98.4% and 91.1% of children, respectively. Breastfeeding indicators showed high prevalence: 90.5% of children were breastfed within the first hour of life, 87.3% received exclusive breastfeeding on the first day at home, and 59.3% were exclusively breastfed up to six months (Table 1).

**Table 1:** Sociodemographic, gestational, birth, breastfeeding, food insecurity, and nutritional characteristics of children attending municipal Early Childhood Education Centers – Paraguaçu, MG, Brazil, 2026.

Variable	N	(%)	95% CI
**Sex**	**139**		
Male	79	56.83	48.17–65.20
Female	60	43.16	34.80–51.83
**Age**	**139**		
Infants from 6 to 11 months	2	1.44	0.17–5.10
Infants from 12 to 23 months	47	33.81	26.01–42.32
Infants from 24 to 35 months	54	38.85	30.70–47.48
Children ≥ 36 months	36	25.90	18.84–34.01
**Race/skin color**	**107**		
White	73	68.23	58.51–76.89
Brown (Mixed race)	24	22.43	14.92–31.51
Black	9	8.41	3.91–15.36
Asian	1	0.93	0.02–5.09
**Socioeconomic Classification (ABEP)**	**137**		
Class A	1	0.73	0.01–3.99
Class B1	13	9.50	5.15–15.68
Class B2	47	34.31	26.41–42.89
Class C1	28	20.44	14.03–28.16
Class C2	33	24.08	17.19–32.13
Class D-E	15	10.94	6.25–17.41
**Brazilian Food Insecurity Scale (EBIA)**	**137**		
Food Security	72	52.55	44.18–60.92
Mild Food Insecurity	49	35.77	27.76–44.40
Moderate Food Insecurity	12	8.76	4.60–14.80
Severe Food Insecurity	4	2.92	0.80–7.30
**Mother’s schooling/Education**	**133**		
Unfinished elementary school (0 to 7 years)	12	9.02	4.74–15.23
Finished elementary school (8 to 10 years)	12	9.02	4.74–15.23
Finished high school (≥11 years)	64	48.13	39.37–56.95
Higher Education	45	33.83	25.86–42.54
**Classification of Newborn according to Gestational Age**	**128**		
Preterm (≤ 37 weeks)	13	10.16	5.51–16.74
Full-term (37 to 41 weeks)	110	85.94	78.68–91.45
Postterm (> 42 weeks)	5	3.90	1.28–8.88
**High-Risk Pregnancy**	**136**		
Yes	15	11.03	6.30–17.53
No	121	88.97	82.46–93.69
**Gestational complications**	**134**		
Yes	33	24.63	17.59–32.81
No	101	75.37	67.18–82.40
**Place of Delivery**	**138**		
Hospital	136	98.56	94.86–99.82
Home birth	1	0.72	0.01–3.97
Other	1	0.72	0.01–3.97
**Type of Delivery**	**131**		
Vaginal	48	36.64	28.39–45.50
Cesarean section	82	62.60	53.71–70.89
Forceps delivery	1	0.76	0.02–4.17
**Postpartum complications**	**132**		
Yes	13	9.85	5.34–16.25
No	119	90.15	83.74–94.65
**Birth Weight**	**133**		
Low birth weight (1500–2499 g)	5	3.76	1.23–8.55
Insufficient birth weight (2500–2999 g)	26	19.55	13.18–27.31
Adequate weight (3000–3999 g)	95	71.43	62.95–78.92
Macrosomia (>4000 g)	7	5.26	2.14–10.54
**Birth length**	**121**		
Low birth length	2	1.65	0.20–5.84
Adequate birth length	119	98.35	94.15–99.79
**Head circumference at birth**	**101**		
Normal	92	91.09	83.75–95.84
Below normal range	9	8.91	4.15–16.24
**Breastfeeding in the first hour of life**	**137**		
Yes	124	90.51	84.32–94.85
No	13	9.49	5.15–15.68
**Exclusive breastfeeding on first day at home**	**134**		
Yes	117	87.31	80.47–92.43
No	17	12.69	7.57–19.53
**Exclusive breastfeeding until 6 months of age**	**135**		
Yes	80	59.26	50.47–67.63
No	55	40.74	32.37–49.53

Source: Study data. ABEP: Brazilian Association of Research Companies. Differences in total sample size (N) are due to missing data across variables.

In the multivariable analysis for HAZ, using a Poisson model with robust variance, no statistically significant and reliable associations were identified between the explanatory variables and the outcome (Table 2).

**Table 2:** Explanatory model for height-for-age (HAZ) in children. Poisson model with robust variance, Nagelkerke R^2^ = 0.3259 – Brazil, 2026.

Explanatory variables	PR^1^	95% CI^2^	p-value^3^
Birth weight (continuous)	0.9996	0.9981–1.0012	0.636
Sex (male vs. female)	5.0438	0.8046–31.6151	0.084
Maternal education			
├─ Finished elementary school	8.0312	0.2769–232.8990	0.225
├─ Finished high school	2.4249	0.0852–68.9697	0.604
└─ Higher education	0.7653	0.0619–9.4529	0.834
Race/skin color (Non-White vs. other)	2.2546	0.5298–9.5941	0.271
Food insecurity (ref.: Food	security)		
├─ Mild	2.2708	0.8398–6.1401	0.106
├─ Moderate	2.2084	0.2901–16.8135	0.444
└─ Severe	0.0000	0.0000–—^*^	**<0.001**
Gestational age (continuous)	1.1782	0.9126–1.5212	0.208
Type of delivery (cesarean vs. vaginal)	0.4319	0.1176–1.5859	0.205
Maternal health condition (categorical)	2.9085	0.8258–10.2447	0.096
High-risk pregnancy (yes vs. no)	0.4461	0.0630–3.1614	0.419
Gestational complications (yes vs. no)	1.4307	0.2949–6.9415	0.656
Head circumference at birth (continuous)	1.0337	0.8672–1.2323	0.711

Notes: ^1^PR: Prevalence ratio.

^2^95% CI: 95% confidence interval.

^3^p-values obtained using the Wald test.

Reference categories (ref.) are indicated for categorical variables. Bold values indicate statistical significance based on both p < 0.05 and 95% CI excluding 1.

^*^Estimate not reliable due to sparse data and model instability; results should not be interpreted.

Birth weight showed an estimate close to the null (PR = 0.9996; 95% CI: 0.9981–1.0012; p = 0.636), indicating no evidence of association with HAZ. Similarly, maternal education, race/skin color, food insecurity (mild and moderate), gestational age, type of delivery, maternal health condition, high-risk pregnancy, gestational complications, and head circumference at birth were not associated with the outcome in the adjusted model.

Some variables, including sex (PR = 5.04; p = 0.084) and maternal health condition (PR = 2.91; p = 0.096), showed borderline p-values; however, their confidence intervals were wide and included the null value, indicating low precision and lack of statistical significance.

Although severe food insecurity presented a statistically significant p-value (p < 0.001), the estimate was unstable (PR = 0.000) due to sparse data and model instability. Therefore, this result is not reliable and should not be interpreted as evidence of association.

Overall, the model explained a moderate proportion of the variability in HAZ (Nagelkerke R^2^ = 0.3259); however, the lack of precise estimates limits the interpretability of the associations.

Socioeconomic status was inversely associated with BAZ. Children from lower socioeconomic classes (C1, C2, and D–E) showed significantly lower prevalence ratios compared to the reference category (Class A), with PR values of 0.14 (95% CI: 0.04–0.49; p = 0.001), 0.09 (95% CI: 0.02–0.58; p = 0.010), and 0.09 (95% CI: 0.01–0.61; p = 0.013), respectively (Table 3).

**Table 3:** Explanatory model for body mass index-for-age (BAZ) in children. Quasi-Poisson model with robust variance, Nagelkerke R^2^ = 0.3518 – Brazil, 2026.

Explanatory variables	PR^1^	95% CI^2^	p-value^3^
Birth weight (continuous)	1.0016	1.0004–1.0028	**0.008**
Sex (male vs. female)	0.6629	0.2573–1.7078	0.394
Race/skin color (Non-White vs. other)	0.5846	0.230–1.4812	0.257
Food insecurity (ref.: Food security)		
├─ Mild	4.1591	1.2746–13.5714	**0.018**
├─ Moderate	9.1878	1.3803–61.1547	**0.021**
└─ Severe	38.8732	4.6400–325.6740	**<0.001**
Socioeconomic Classification (ABEP) (ref.: Class A)			
├─ Class B1	0.5335	0.1361–2.0912	0.367
├─ Class B2	0.4830	0.1652–1.4119	0.183
├─ Class C1	0.1416	0.0411–0.4873	**0.001**
├─ Class C2	0.0949	0.0156–0.5777	**0.010**
└─ Class D-E	0.0857	0.0120–0.6083	**0.013**
Gestational age (continuous)	0.8565	0.6745–1.0875	0.203
Type of delivery (cesarean vs. vaginal)	0.5773	0.2641–1.2618	0.168
High-risk pregnancy (yes vs. no)	3.0420	1.1698–7.9107	**0.022**
Head circumference at birth (continuous)	0.6829	0.4586–1.0169	0.060

Notes: ^1^PR: Prevalence ratio.

^2^95% CI: 95% confidence interval.

^3^p-values obtained using the Wald test.

Reference categories (ref.) are indicated for categorical variables. Bold values indicate statistical significance based on both p < 0.05 and 95% CI excluding 1.

High-risk pregnancy was positively associated with BAZ (PR = 3.04; 95% CI: 1.17–7.91; p = 0.022), indicating a higher prevalence of the outcome among these children.

Other variables, including sex, race/skin color, gestational age, type of delivery, and head circumference at birth, were not significantly associated with BAZ in the adjusted model, although head circumference showed borderline statistical significance (p = 0.060).

Overall, the model explained a moderate proportion of the variability in BAZ (Nagelkerke R^2^ = 0.3518), indicating acceptable model fit.

## DISCUSSION

Given the cross-sectional design, the results must be interpreted with caution regarding temporality and causality. The assessment and monitoring of children’s nutritional status are essential for identifying nutritional disorders and guiding health promotion and disease prevention strategies, especially during early childhood, a period marked by rapid growth and high nutritional vulnerability. Anthropometric indicators are widely recognized as sensitive markers of population health and quality of life, reflecting both biological conditions and the socioeconomic context in which children are embedded^(16,17)^.

In Brazil, data from the National Survey on Child Nutrition (*ENANI-2019*) indicate a prevalence of short stature of 7.0% among children under five years of age, increasing to 10.2% among those aged 12 to 23 months. The prevalence of excess weight in this population was 10.1%, reaching 13.7% in the same age group^(18)^. In contrast, the present study identified substantially higher prevalences of short stature (22.6%) and overweight (34.4%), indicating a less favorable nutritional profile in the evaluated population compared to national averages. These differences may reflect local socioeconomic conditions, dietary patterns, and contextual vulnerabilities not fully captured in national surveys.

Although national surveillance data have shown a reduction in the prevalence of overweight among children under five years of age between 2008 and 2018, as reported by Vasconcelos et al.^(19)^, excess weight in childhood remains a major public health concern. The coexistence of growth deficits and excess weight observed in this study reinforces the ongoing nutritional transition in Brazil, particularly in socially vulnerable populations, where structural inequalities contribute to the simultaneous presence of undernutrition and overweight.

Food and nutrition insecurity was identified in nearly half of the households, a prevalence similar to that reported by Gomes and Gubert^(20)^ using data from the PNDS-2006. This finding is also consistent with the worsening scenario described by the II National Survey on Food Insecurity in the Context of the COVID-19 Pandemic (II VIGISAN)^(21,22)^. In the present study, food insecurity was significantly associated with BMI-for-age, with higher prevalence ratios observed across increasing levels of severity. This finding suggests a potential relationship between household food access and nutritional outcomes, possibly mediated by reduced diet quality and increased consumption of ultra-processed foods, which are known contributors to excess weight in childhood.

Despite advances in public policies and social protection programs, food and nutrition insecurity remains a major challenge in Brazil, reflecting persistent socioeconomic inequalities. These findings highlight the importance of strengthening intersectoral strategies aimed at improving access to adequate and healthy food, promoting exclusive breastfeeding, and ensuring appropriate complementary feeding practices during early childhood^(23)^.

Regarding birth characteristics, the proportion of cesarean deliveries observed in this study was higher than that reported in national surveys such as *PNDS*-2006^(24)^. Although causal inferences cannot be made, this finding reflects the persistently high cesarean rates in Brazil and underscores the need for continued monitoring, given their potential implications for maternal and child health outcomes.

Breastfeeding indicators in the studied population were more favorable than those reported in national and regional surveys. The high prevalence of breastfeeding in the first hour of life and exclusive breastfeeding practices may reflect improvements in neonatal care and local health service performance, which are known to have positive effects on child growth and development^(25,26)^.

Adequate prenatal care is consistently associated with improved pregnancy outcomes, including appropriate birth weight and gestational age. Although most mothers in this study had access to prenatal care, the presence of gestational complications and high-risk pregnancies highlights the need for continuous monitoring and timely interventions to mitigate potential adverse effects on child health^(27,28)^.

Child growth results from a complex interplay of genetic, intrauterine, and postnatal factors. In the present study, multivariable analyses identified significant associations between BMI-for-age and selected variables, including birth weight, food insecurity, socioeconomic status, and high-risk pregnancy. Birth weight showed a positive association with BMI-for-age, although with small magnitude, suggesting that higher birth weight may be related to a higher prevalence of excess weight in early childhood. Additionally, children exposed to food insecurity presented higher prevalence ratios of BMI-for-age alteration, reinforcing the role of social determinants in shaping nutritional outcomes.

Socioeconomic status was inversely associated with BMI-for-age, with lower socioeconomic classes presenting lower prevalence ratios compared to the highest class. This finding may reflect complex and context-specific patterns of nutritional transition, in which excess weight is not uniformly distributed across socioeconomic strata. High-risk pregnancy was also associated with BMI-for-age, suggesting that adverse gestational conditions may have implications for early growth trajectories^(29,30,31,32,33)^.

In contrast, no consistent associations were observed between the evaluated variables and height-for-age. Overall, the findings indicate that anthropometric outcomes may be differentially influenced by early-life and contextual factors, particularly in relation to excess weight.

These findings should be interpreted with caution, as the wide confidence intervals observed across several estimates indicate limited precision. Additionally, the cross-sectional design does not allow establishing temporal or causal relationships between exposures and outcomes. Residual confounding, small subgroup sizes, and variability in exposure distribution may also have influenced the results.

Finally, due to the cross-sectional nature of the study, the findings should be considered exploratory and hypothesis-generating, reinforcing the need for longitudinal studies to better elucidate the role of early-life conditions and social determinants in shaping child growth trajectories.

### Study Limitations

Some limitations of this study should be acknowledged. Due to its cross-sectional design, it is not possible to establish temporal or causal relationships between nutritional status and the exposure variables analyzed. In addition, the study was conducted in a single municipality and included children enrolled in municipal daycare centers, which may limit the generalizability of the findings to other populations and contexts.

Non-participation due to refusal or incomplete data may have introduced selection bias, although the participation rate was high (79.8%). Furthermore, the small number of participants in certain neonatal subgroups, particularly preterm birth and low birth weight, may have reduced statistical power, contributing to the wide confidence intervals observed in some multivariable estimates. Although Fisher’s exact test was applied when appropriate, small cell frequencies may still limit the robustness of some comparisons.

Information on gestational history and socioeconomic conditions was obtained through reports from parents or legal guardians and may therefore be subject to recall bias. Nevertheless, the use of standardized and validated instruments, along with the consistency of the findings with existing literature, supports the internal validity of the study.

## CONCLUSION

The present study identified a high prevalence of short stature and excess weight among children enrolled in municipal Early Childhood Education Centers, with proportions exceeding those reported in national surveys.

In the adjusted analyses, body mass index-for-age was associated with birth weight, food insecurity, socioeconomic classification, and high-risk pregnancy, highlighting the influence of both early-life conditions and social determinants on excess weight in childhood. In contrast, no statistically significant associations were observed for height-for-age.

The high prevalence of food and nutrition insecurity further underscores the persistence of social and economic vulnerabilities that may adversely affect child health and development. These findings reinforce the importance of continuous monitoring of nutritional status in early childhood and the strengthening of integrated public health policies addressing both biological and social determinants, particularly in socially vulnerable contexts.

## Data Availability

The entire dataset supporting the results of this study is available upon request to the corresponding author.
