# Supplementary material for: Association between neonatal conditions and anthropometric indicators among children attending daycare centers in Southern Brazil
Source: Rev Esc Enferm USP. 2026 Jul 24;60:e20250598. doi: 10.1590/1980-220X-REEUSP-2025-0598en (PMC13404304; doi:10.1590/1980-220X-REEUSP-2025-0598en)
Supplement: Translation

# Translation: Asociación entre las condiciones neonatales y los indicadores antropométricos en niños que asisten a guarderías en el sur de Brasil

**DOI:** 10.1590/1980-220X-REEUSP-2025-0598es

**Authors:** Luiz Felipe de Paiva Lourenção, Lara Campos Borim, Laura Thaciane Rodrigues Cassiano, Roseli Oselka Saccardo Sarni, Rosangela Silva

**Affiliations:** 1 Universidade Federal de Alfenas, Faculdade de Nutrição, Alfenas, MG, Brasil.; 2 Universidade Federal de São Paulo, Escola Paulista de Medicina, São Paulo, SP, Brasil.; 3 Centro Universitário FMABC, Santo André, SP, Brasil.

**Keywords:** Nutrición del Niño, Crecimiento y Desarrollo, Seguridad Alimentaria, Antropometría

## Abstract

**Objetivo:**

Investigar la asociación entre las condiciones neonatales y los indicadores antropométricos, y describir la inseguridad alimentaria entre los niños que asisten a guarderías.

**Métodos:**

Estudio transversal con 139 niños matriculados en centros de educación infantil en el sur de Minas Gerais, Brasil. Se recolectaron datos sobre afecciones neonatales, características socioeconómicas, inseguridad alimentaria y estado nutricional. La estatura para la edad (HAZ) y el índice de masa corporal para la edad (BAZ) se clasificaron según los estándares de la Organización Mundial de la Salud. Se utilizó una regresión de Poisson con varianza robusta para estimar las razones de prevalencia (RP).

**Resultados:**

La prevalencia de sobrepeso fue del 34,4 %, y se observó baja estatura en el 22,6 % de los niños. La prevalencia de sobrepeso fue del 34,4 % y la de baja estatura del 22,6 %. El BAZ se asoció con el peso al nacer (RP = 1,0016; IC del 95 %: 1,0004–1,0028), la inseguridad alimentaria, el estatus socioeconómico y el embarazo de alto riesgo. Los niveles más altos de inseguridad alimentaria mostraron RP más elevados para el BAZ, mientras que las clases socioeconómicas más bajas se asociaron de manera inversa. No se observaron asociaciones para el HAZ.

**Conclusión:**

El BAZ se asoció con factores perinatales y socioeconómicos, lo que pone de relieve los determinantes del exceso de peso en las primeras etapas de la vida y la influencia de la inseguridad alimentaria, lo que refuerza la necesidad de intervenciones tempranas.

## INTRODUCCIÓN

Las condiciones adecuadas durante el embarazo y el parto, la lactancia materna exclusiva hasta los seis meses de edad y una alimentación complementaria saludable desempeñan un papel esencial a lo largo de toda la vida, particularmente en la primera infancia, contribuyendo al crecimiento y desarrollo adecuados, a la formación de hábitos saludables y a la promoción de la salud^(1,2)^.

Durante los dos primeros años de vida, la elección de los alimentos requiere una atención especial, ya que este período es crítico para el establecimiento de los hábitos alimentarios. Las dietas basadas en alimentos frescos o mínimamente procesados deben constituir la base de la alimentación tanto del niño como de la familia. En este contexto, la diversidad y la forma en que se ofrecen los alimentos influyen en el desarrollo de las preferencias gustativas, la relación de los niños con la comida y su estado nutricional. Las estrategias de atención de salud y nutrición implementadas durante este período pueden tener repercusiones a lo largo de toda la vida^(3)^. Por lo tanto, el estado nutricional de los niños se considera un indicador sensible de la salud y la calidad de vida, que refleja el modelo de desarrollo de una sociedad determinada^(4)^.

Estudios recientes indican que la alimentación de los niños se caracteriza por una baja diversidad alimentaria, un consumo reducido de alimentos frescos o mínimamente procesados y una exposición temprana a alimentos ultraprocesados, particularmente en hogares de bajos ingresos y con bajo nivel educativo. Este patrón alimentario se asocia con un mayor riesgo de sobrepeso, obesidad y enfermedades no transmisibles (ENT) en etapas posteriores de la vida^(5,6,7)^. Los alimentos ultraprocesados son formulaciones industriales de baja calidad nutricional, caracterizadas por una alta densidad energética, niveles elevados de grasas, azúcares y sodio, y un bajo contenido de fibra, como resultado de múltiples etapas de procesamiento industrial y la adición de sustancias para mejorar la palatabilidad y la vida útil^(8)^.

Las condiciones al nacer desempeñan un papel fundamental en el desarrollo de trastornos nutricionales, incluyendo la desnutrición y el exceso de peso. El peso al nacer es un indicador importante del estado nutricional neonatal, y el bajo peso al nacer (BPN) se reconoce como un factor de riesgo de morbilidad y mortalidad infantil y posneonatal, así como de retraso en el desarrollo infantil^(9,10)^.

Una dieta rica en alimentos ultraprocesados durante la primera infancia afecta negativamente al crecimiento y al desarrollo y contribuye al sobrepeso en esta población. Este patrón alimentario se ha relacionado con consecuencias para la salud a largo plazo, como la obesidad, la diabetes, la hipertensión y la dislipidemia^(11,12)^. Aunque estudios previos han explorado el papel de las condiciones de la primera infancia y los patrones alimentarios en el crecimiento infantil, la evidencia sobre la asociación entre las condiciones neonatales y los indicadores antropométricos en niños que asisten a guarderías, particularmente en pequeños municipios brasileños, sigue siendo limitada. Por lo tanto, este estudio tuvo como objetivo investigar la relación entre las condiciones neonatales y los indicadores antropométricos y describir la seguridad alimentaria y nutricional entre los niños inscritos en guarderías.

## MÉTODO

### Tipo de Estudio

Este fue un estudio transversal realizado entre niños matriculados en Centros de Educación Infantil municipales en un municipio del sur de Minas Gerais, Brasil.

### Entorno del Estudio

El estudio se llevó a cabo en tres Centros de Educación Infantil (CEI) municipales ubicados en el mismo municipio. La recolección de datos se llevó a cabo entre abril y julio de 2022. Los CEI funcionan a tiempo completo y ofrecen cuatro comidas diarias (desayuno, almuerzo, merienda y cena), diseñadas para cubrir al menos el 70 % de las necesidades nutricionales diarias de energía, macronutrientes y micronutrientes prioritarios (vitaminas A y C, calcio y hierro).

### Participantes y Muestra

Se consideró que todos los niños matriculados en los Centros de Educación Infantil seleccionados durante el período de recolección de datos eran elegibles para participar, lo que caracteriza un enfoque censal en lugar de un muestreo probabilístico.

Un total de 174 niños cumplieron con los criterios de elegibilidad. De ellos, 23 no participaron debido a la negativa de los padres o tutores legales a dar su consentimiento informado, y 12 fueron excluidos debido a datos incompletos en variables clave del estudio. Así, la muestra analítica final consistió en 139 niños (79,8 % de la población elegible). No se observaron diferencias significativas en la distribución por edad y sexo entre participantes y no participantes.

La participación fue voluntaria y dependía de la autorización de los padres, lo que pudo haber contribuido a la no participación. Se excluyó del estudio a los niños con discapacidades neurológicas y/o aquellos que tomaban medicamentos corticosteroides. Por lo tanto, la muestra final se obtuvo de la población elegible durante el período de estudio y no se basó en un cálculo previo del tamaño de la muestra.

### Variables

Los resultados principales del estudio fueron los indicadores del estado nutricional, evaluados mediante los escores Z de estatura para la edad (HAZ) e índice de masa corporal para la edad (BAZ), clasificados según los estándares de referencia de la Organización Mundial de la Salud (OMS) (escores Z < –2 indican déficit y > +2 indican exceso de peso).

Las variables de exposición incluyeron la edad gestacional, el peso al nacer, la altura al nacer, las prácticas de lactancia materna y la seguridad alimentaria y nutricional del hogar. También se recolectaron variables sociodemográficas para caracterizar a la población del estudio.

### Procedimientos de Recolección de Datos

Los datos se recopilaron mediante un cuestionario estructurado y estandarizado administrado a los padres o tutores legales. El cuestionario incluía información sobre el historial gestacional, las condiciones neonatales y de nacimiento, las prácticas de lactancia materna y las características socioeconómicas.

El estatus socioeconómico se evaluó utilizando los Criterios de Clasificación Económica Brasileños establecidos por la Asociación Brasileña de Empresas de Investigación (ABEP)^(13)^.

Para minimizar el sesgo de información, la recolección de datos fue realizada por investigadores capacitados utilizando instrumentos y procedimientos estandarizados.

### Evaluación del Estado Nutricional

Las mediciones antropométricas de peso y estatura se obtuvieron en los Centros de Educación Infantil siguiendo los procedimientos estandarizados recomendados por la Organización Mundial de la Salud (OMS). Las mediciones de peso y estatura se utilizaron para calcular el HAZ y el BAZ, clasificados según los estándares de referencia de la OMS. El procesamiento y la clasificación de los datos se realizaron utilizando el software Anthro® de la OMS^(14)^.

### Evaluación de la Seguridad Alimentaria y Nutricional

La seguridad alimentaria y nutricional se evaluó utilizando la Escala Brasileña de Inseguridad Alimentaria (EBIA), un instrumento psicométrico validado compuesto por 14 preguntas dicotómicas (“sí” o “no”) que evalúan el acceso de los hogares a los alimentos basándose en la percepción y la experiencia de inseguridad alimentaria durante los tres meses anteriores^(15)^.

### Análisis Estadístico

Se realizaron análisis descriptivos para caracterizar a la población del estudio, utilizando frecuencias absolutas y relativas para las variables categóricas, junto con sus respectivos intervalos de confianza del 95 % (IC del 95 %), calculados suponiendo una distribución binomial. Las variables continuas se describieron mediante medias y desviaciones estándar, de acuerdo con su distribución.

Se realizaron análisis bivariados para explorar las relaciones entre las variables de exposición y los resultados. Se aplicó la prueba de chi-cuadrado de Pearson cuando se cumplían los supuestos, y se utilizó la prueba exacta de Fisher en los casos de frecuencias celulares esperadas pequeñas para garantizar la validez de la inferencia estadística.

Se realizaron análisis multivariables utilizando modelos lineales generalizados para estimar las razones de prevalencia (RP) y los correspondientes intervalos de confianza del 95 %. Teniendo en cuenta el diseño transversal y la naturaleza no rara de los resultados, se adoptó la regresión de Poisson con varianza robusta como estrategia de modelización principal.

Para el resultado BAZ, se utilizó un modelo de Poisson con varianza robusta, suponiendo una distribución aproximadamente equidispersa. Para el resultado HAZ, se aplicó un modelo cuasi-Poisson para tener en cuenta los parámetros de dispersión diferentes de la unidad, mejorando la adecuación de la estimación de la varianza.

Las variables con p < 0,20 en los análisis bivariados se incluyeron en los modelos multivariables, y la significación estadística se definió como p < 0,05. Las estimaciones del modelo se interpretaron con cautela cuando los intervalos de confianza eran amplios o incluían el valor nulo, lo que indicaba una precisión limitada.

Todos los análisis estadísticos se realizaron utilizando el software R, versión 4,5,2.

### Aspectos Éticos

El estudio fue aprobado por el Comité de Ética en Investigación de la Universidade Federal de São Paulo (UNIFESP), bajo el dictamen n.º 3,936,371, de fecha 2020. Todos los procedimientos se llevaron a cabo de acuerdo con las directrices éticas y las normas reglamentarias para la investigación con seres humanos, según lo establecido por las Resoluciones del Consejo Nacional de Salud de Brasil n.º 466/2012 y n.º 510/2016. Los padres o tutores legales recibieron información detallada sobre los objetivos y procedimientos del estudio y dieron su consentimiento informado por escrito antes de la participación de los niños.

## RESULTADOS

Se incluyó a un total de 139 niños; la mayoría eran varones (56,8 %; IC del 95 %: 48,2–65,2) y tenían entre 24 y 35 meses de edad (38,9 %; IC del 95 %: 30,7–47,5). La mayoría de los niños fueron clasificados como blancos (68,2 %), y la clase socioeconómica predominante fue la B2 (34,3 %). Se observó inseguridad alimentaria y nutricional en el 47,5 % de los hogares, predominantemente en el nivel leve (35,8 %), mientras que las formas moderadas y graves fueron menos frecuentes (8,8 % y 2,9 %, respectivamente). Se observó seguridad alimentaria en el 52,6 % de los hogares.

La mayoría de las madres habían terminado la escuela secundaria (48,1 %), y el 33,8 % tenía educación superior. Se produjeron partos prematuros en el 10,2 % de los casos, y el 11,0 % de los embarazos se clasificaron como de alto riesgo. Se reportaron complicaciones gestacionales en el 24,6 % de los casos. Casi todos los partos se produjeron en entornos hospitalarios (98,6 %), siendo la cesárea el modo predominante (62,6 %). Se reportaron complicaciones posparto en el 9,9 % de los casos. La mayoría de los niños nacieron con un peso adecuado al nacer (71,4 %), mientras que el bajo peso al nacer y la macrosomía fueron menos frecuentes (3,8 % y 5,3 %, respectivamente). Se observó una estatura al nacer adecuada y un perímetro cefálico normal en el 98,4 % y el 91,1 % de los niños, respectivamente. Los indicadores de lactancia materna mostraron una alta prevalencia: el 90,5 % de los niños fueron amamantados en la primera hora de vida, el 87,3 % recibieron lactancia materna exclusiva el primer día en casa y el 59,3 % fueron amamantados exclusivamente hasta los seis meses (Tabla 1).

**Tabla 1:** Características sociodemográficas, gestacionales, del parto, de la lactancia materna, de la inseguridad alimentaria y nutricionales de los niños que asisten a los Centros Municipales de Educación Infantil – Paraguaçu, MG, Brasil, 2026.

Variable	N	(%)	95% IC
**Sexo**	**139**		
Masculino	79	56,83	48,17–65,20
Femenino	60	43,16	34,80–51,83
**Edad**	**139**		
Bebés de 6 a 11 meses	2	1,44	0,17–5,10
Bebés de 12 a 23 meses	47	33,81	26,01–42,32
Bebés de 24 a 35 meses	54	38,85	30,70–47,48
Niños ≥ 36 meses	36	25,90	18,84–34,01
**Raza/Color de piel**	**107**		
Blanca	73	68,23	58,51–76,89
Mestizo	24	22,43	14,92–31,51
Negro	9	8,41	3,91–15,36
Asiático	1	0,93	0,02–5,09
**Clasificación socioeconómica (ABEP)**	**137**		
Clase A	1	0,73	0,01–3,99
Clase B1	13	9,50	5,15–15,68
Clase B2	47	34,31	26,41–42,89
Clase C1	28	20,44	14,03–28,16
Clase C2	33	24,08	17,19–32,13
Clase D-E	15	10,94	6,25–17,41
**Escala Brasileña de Inseguridad Alimentaria (EBIA)**	**137**		
Seguridad alimentaria	72	52,55	44,18–60,92
Inseguridad alimentaria leve	49	35,77	27,76–44,40
Inseguridad alimentaria moderada	12	8,76	4,60–14,80
Inseguridad alimentaria grave	4	2,92	0,80–7,30
**Formación académica de la madre**	**133**		
Sin estudios primarios completos (0 a 7 años)	12	9,02	4,74–15,23
Estudios primarios completos (8 a 10 años)	12	9,02	4,74–15,23
Estudios secundarios completos (≥11 años)	64	48,13	39,37–56,95
Educación superior	45	33,83	25,86–42,54
**Clasificación de los recién nacidos según la edad gestacional**	**128**		
Prematuro (≥ 37 semanas)	13	10,16	5,51–16,74
A término (37 a 41 semanas)	110	85,94	78,68–91,45
Post término (> 42 semanas)	5	3,90	1,28–8,88
**Embarazo de alto riesgo**	**136**		
Sí	15	11,03	6,30–17,53
No	121	88,97	82,46–93,69
**Complicaciones gestacionales**	**134**		
Sí	33	24,63	17,59–32,81
No	101	75,37	67,18–82,40
**Lugar del parto**	**138**		
Hospital	136	98,56	94,86–99,82
Parto en casa	1	0,72	0,01–3,97
Otro	1	0,72	0,01–3,97
**Tipo de parto**	**131**		
Vaginal	48	36,64	28,39–45,50
Cesárea	82	62,60	53,71–70,89
Parto con fórceps	1	0,76	0,02–4,17
**Complicaciones posparto**	**132**		
Sí	13	9,85	5,34–16,25
No	119	90,15	83,74–94,65
**Peso al nacer**	**133**		
Bajo peso al nacer (1500–2499 g)	5	3,76	1,23–8,55
Peso al nacer insuficiente (2500–2999 g)	26	19,55	13,18–27,31
Peso adecuado (3000–3999 g)	95	71,43	62,95–78,92
Macrosomía (>4000 g)	7	5,26	2,14–10,54
**Altura al nacer**	**121**		
Altura al nacer baja	2	1,65	0,20–5,84
Altura al nacer adecuada	119	98,35	94,15–99,79
**Perímetro cefálico al nacer**	**101**		
Normal	92	91,09	83,75–95,84
Por debajo del rango normal	9	8,91	4,15–16,24
**Lactancia materna en la primera hora de vida**	**137**		
Sí	124	90,51	84,32–94,85
No	13	9,49	5,15–15,68
**Lactancia materna exclusiva el primer día en casa**	**134**		
Sí	117	87,31	80,47–92,43
No	17	12,69	7,57–19,53
**Lactancia materna exclusiva hasta los 6 meses de edad**	**135**		
Sí	80	59,26	50,47–67,63
No	55	40,74	32,37–49,53

Fuente: Datos del estudio. ABEP: Asociación Brasileña de Empresas de Investigación. Las diferencias en el tamaño total de la muestra (N) se deben a la ausencia de datos en algunas variables.

En el análisis multivariable para el HAZ, utilizando un modelo de Poisson con varianza robusta, no se identificaron asociaciones estadísticamente significativas ni fiables entre las variables explicativas y el resultado (Tabla 2).

**Tabla 2:** Modelo explicativo para la estatura para la edad (HAZ) en niños. Modelo de Poisson con varianza robusta, R2 de Nagelkerke = 0,3259 – Brasil, 2026.

Variables explicativas	RP^1^	95% IC^2^	p-valor^3^
Peso al nacer (continuo)	0,9996	0,9981–1,0012	0,636
Sexo (masculino / femenino)	5,0438	0,8046–31,6151	0,084
Educación materna			
├─ Ha terminado la escuela primaria	8,0312	0,2769–232,8990 0,225
├─ Ha terminado la escuela secundaria	2,4249	0,0852–68,9697	0,604
└─ Educación superior	0,7653	0,0619–9,4529	0,834
Raza/color de piel (no blancos / otros)	2,2546	0,5298–9,5941	0,271
Inseguridad alimentaria (ref: Seguridad alimentaria)	
├─ Leve	2,2708	0,8398–6,1401	0,106
├─ Moderado	2,2084	0,2901–16,8135	0,444
└─ Grave	0,0000	0,0000–—^*^	<**0,001**
Edad gestacional (variable continua)	1,1782	0,9126–1,5212	0,208
Tipo de parto (por cesárea / vaginal)	0,4319	0,1176–1,5859	0,205
Estado de salud de la madre (variable categórica)	2,9085	0,8258–10,2447	0,096
Embarazo de alto riesgo (sí / no)	0,4461	0,0630–3,1614	0,419
Complicaciones gestacionales (sí / no)	1,4307	0,2949–6,9415	0,656
Perímetro cefálico al nacer (variable continua)	1,0337	0,8672–1,2323	0,711

Notas: ^1^RP: Razón de prevalencia.

^2^IC del 95 %: Intervalo de confianza del 95 %.

^3^P-valores obtenidos mediante la prueba de Wald.

Se indican las categorías de referencia (ref.) para las variables categóricas. Los valores en negrita indican significación estadística basada tanto en p < 0,05 como en un IC del 95 % que excluye el valor 1.

^*^Estimación no fiable debido a la escasez de datos y a la inestabilidad del modelo; no se deben interpretar los resultados.

El peso al nacer mostró una estimación cercana a la nula (RP = 0,9996; IC del 95 %: 0,9981–1,0012; p = 0,636), lo que indica que no hay evidencia de asociación con el HAZ. De manera similar, la educación materna, la raza/color de piel, la inseguridad alimentaria (leve y moderada), la edad gestacional, el tipo de parto, el estado de salud materna, el embarazo de alto riesgo, las complicaciones gestacionales y el perímetro cefálico al nacer no se asociaron con el resultado en el modelo ajustado.

Algunas variables, entre ellas el sexo (RP = 5,04; p-valor = 0,084) y el estado de salud materna (RP = 2,91; p-valor = 0,096), mostraron p-valores límite; sin embargo, sus intervalos de confianza eran amplios e incluían el valor nulo, lo que indica baja precisión y falta de significación estadística.

Aunque la inseguridad alimentaria grave presentó un p-valor estadísticamente significativo (p < 0,001), la estimación fue inestable (RP = 0,000) debido a la escasez de datos y a la inestabilidad del modelo. Por lo tanto, este resultado no es confiable y no debe interpretarse como evidencia de asociación.

En general, el modelo explicó una proporción moderada de la variabilidad en el HAZ (R^2^ de Nagelkerke = 0,3259); sin embargo, la falta de estimaciones precisas limita la interpretabilidad de las asociaciones.

El estatus socioeconómico se asoció de forma inversa con el BAZ. Los niños de las clases socioeconómicas más bajas (C1, C2 y D–E) mostraron razones de prevalencia significativamente más bajas en comparación con la categoría de referencia (Clase A), con valores de RP de 0,14 (IC del 95 %: 0,04–0,49; p-valor = 0,001), 0,09 (IC del 95 %: 0,02–0,58; p = 0,010) y 0,09 (IC del 95 %: 0,01–0,61; p = 0,013), respectivamente.

El embarazo de alto riesgo se asoció positivamente con el BAZ (RP = 3,04; IC del 95 %: 1,17–7,91; p = 0,022), lo que indica una mayor prevalencia del resultado entre estos niños. Otras variables, como el sexo, la raza/color de piel, la edad gestacional, el tipo de parto y el perímetro cefálico al nacer, no se asociaron significativamente con el BAZ en el modelo ajustado, aunque el perímetro cefálico mostró una significación estadística límite (p = 0,060).

En general, el modelo explicó una proporción moderada de la variabilidad en el BAZ (R^2^ de Nagelkerke = 0,3518), lo que indica un ajuste aceptable del modelo (Tabla 3).

**Tabla 3:** Modelo explicativo del índice de masa corporal para la edad (BAZ) en niños. Modelo cuasi-Poisson con varianza robusta, R2 de Nagelkerke = 0,3518 – Brasil, 2026.

Variables explicativas	RP^1^	95% IC^2^	p-valor^3^
Peso al nacer (variable continua)	1,0016	1,0004–1,0028	0,008
Sexo (masculino frente a femenino)	0,6629	0,2573–1,7078	0,394
Raza/color de piel (no blanco frente a otros)	0,5846	0,230–1,4812	0,257
Inseguridad alimentaria (ref: Seguridad alimentaria)	
├─ Leve	4,1591	1,2746–13,5714	**0,018**
├─ Moderado	9,1878	1,3803–61,1547	**0,021**
└─ Grave	38,8732	4,6400–325,6740	<**0,001**
Clasificación socioeconómica (ABEP) (ref.: Clase A)			
├─ Clase B1	0,5335	0,1361–2,0912	0,367
├─ Clase B2	0,4830	0,1652–1,4119	0,183
├─ Clase C1	0,1416	0,0411–0,4873	**0,001**
├─ Clase C2	0,0949	0,0156–0,5777	**0,010**
└─ Clase D-E	0,0857	0,0120–0,6083	**0,013**
Edad gestacional (variable continua)	0,8565	0,6745–1,0875	0,203
Tipo de parto (por cesárea / vaginal)	0,5773	0,2641–1,2618	0,168
Embarazo de alto riesgo (sí / no)	3,0420	1,1698–7,9107	**0,022**
Perímetro cefálico al nacer (variable continua)	0,6829	0,4586–1,0169	0,060

Notas: ^1^RP: Razón de prevalencia.

^2^IC del 95 %: Intervalo de confianza del 95 %.

^3^P-valores obtenidos mediante la prueba de Wald.

Se indican las categorías de referencia (ref.) para las variables categóricas. Los valores en negrita indican significación estadística basada tanto en p < 0,05 como en un IC del 95 % que excluye el valor 1.

## DISCUSIÓN

Dado el diseño transversal, los resultados deben interpretarse con cautela en lo que respecta a la temporalidad y la causalidad. La evaluación y el monitoreo del estado nutricional de los niños son esenciales para identificar trastornos nutricionales y orientar las estrategias de promoción de la salud y prevención de enfermedades, especialmente durante la primera infancia, un período caracterizado por un rápido crecimiento y una elevada vulnerabilidad nutricional. Los indicadores antropométricos son ampliamente reconocidos como marcadores sensibles de la salud de la población y la calidad de vida, ya que reflejan tanto las condiciones biológicas como el contexto socioeconómico en el que se encuentran los niños^(16,17)^.

En Brasil, los datos de la Encuesta Nacional de Nutrición Infantil (*ENANI-2019*) indican una prevalencia de estatura baja del 7,0 % entre los niños menores de cinco años, que aumenta al 10,2 % entre los de 12 a 23 meses. La prevalencia de sobrepeso en esta población fue del 10,1 %, alcanzando el 13,7 % en el mismo grupo de edad^(18)^. En contraste, el presente estudio identificó prevalencias sustancialmente más altas de baja estatura (22,6 %) y sobrepeso (34,4 %), lo que indica un perfil nutricional menos favorable en la población evaluada en comparación con los promedios nacionales. Estas diferencias pueden reflejar condiciones socioeconómicas locales, patrones alimentarios y vulnerabilidades contextuales que no se captan plenamente en las encuestas nacionales.

Aunque los datos de vigilancia nacional han mostrado una reducción en la prevalencia del sobrepeso entre los niños menores de cinco años entre 2008 y 2018, tal como lo informaron Vasconcelos et al.^(19)^, el sobrepeso en la infancia sigue siendo una preocupación importante de salud pública. La coexistencia de déficits de crecimiento y sobrepeso observada en este estudio refuerza la transición nutricional en curso en Brasil, particularmente en las poblaciones socialmente vulnerables, donde las desigualdades estructurales contribuyen a la presencia simultánea de desnutrición y sobrepeso.

Se identificó inseguridad alimentaria y nutricional en casi la mitad de los hogares, una prevalencia similar a la reportada por Gomes y Gubert^(20)^ utilizando datos del PNDS-2006. Este hallazgo también concuerda con el escenario de empeoramiento descrito por la II Encuesta Nacional sobre Inseguridad Alimentaria en el Contexto de la Pandemia de COVID-19 (II VIGISAN)^(21,22)^. En el presente estudio, la inseguridad alimentaria se asoció significativamente con el IMC para la edad, observándose mayores índices de prevalencia a medida que aumentaban los niveles de gravedad. Este hallazgo sugiere una posible relación entre el acceso de los hogares a los alimentos y los resultados nutricionales, posiblemente mediada por una menor calidad de la dieta y un mayor consumo de alimentos ultraprocesados, que se sabe que contribuyen al sobrepeso en la infancia.

A pesar de los avances en las políticas públicas y los programas de protección social, la inseguridad alimentaria y nutricional sigue siendo un gran desafío en Brasil, lo que refleja las persistentes desigualdades socioeconómicas. Estos hallazgos resaltan la importancia de fortalecer las estrategias intersectoriales destinadas a mejorar el acceso a alimentos adecuados y saludables, promover la lactancia materna exclusiva y garantizar prácticas adecuadas de alimentación complementaria durante la primera infancia^(23)^.

En cuanto a las características del parto, la proporción de partos por cesárea observada en este estudio fue mayor que la reportada en encuestas nacionales como la *PNDS*-2006^(24)^. Aunque no se pueden establecer inferencias causales, este hallazgo refleja las tasas de cesáreas persistentemente altas en Brasil y subraya la necesidad de monitorear continuamente, dadas sus posibles implicaciones para los resultados de salud materno-infantil.

Los indicadores de lactancia materna en la población estudiada fueron más favorables que los reportados en encuestas nacionales y regionales. La alta prevalencia de la lactancia materna en la primera hora de vida y las prácticas de lactancia materna exclusiva pueden reflejar mejoras en la atención neonatal y el desempeño de los servicios de salud locales, que se sabe que tienen efectos positivos en el crecimiento y el desarrollo infantil^(25,26)^.

La atención prenatal adecuada se asocia sistemáticamente con mejores resultados del embarazo, incluyendo un peso al nacer y una edad gestacional adecuados. Aunque la mayoría de las madres de este estudio tuvieron acceso a la atención prenatal, la presencia de complicaciones gestacionales y embarazos de alto riesgo pone de relieve la necesidad de monitorear continuamente y realizar intervenciones oportunas para mitigar los posibles efectos adversos sobre la salud infantil^(27,28)^.

El crecimiento infantil es el resultado de una compleja interacción de factores genéticos, intrauterinos y posnatales. En el presente estudio, los análisis multivariables identificaron asociaciones significativas entre el IMC para la edad y variables seleccionadas, incluyendo el peso al nacer, la inseguridad alimentaria, el estatus socioeconómico y el embarazo de alto riesgo. El peso al nacer mostró una asociación positiva con el IMC para la edad, aunque de pequeña magnitud, lo que sugiere que un mayor peso al nacer podría estar relacionado con una mayor prevalencia de sobrepeso en la primera infancia. Además, los niños expuestos a la inseguridad alimentaria presentaron mayores razones de prevalencia de alteraciones en el IMC para la edad, lo que refuerza el papel de los determinantes sociales en la configuración de los resultados nutricionales.

El estatus socioeconómico se asoció inversamente con el IMC para la edad, y las clases socioeconómicas más bajas presentaron índices de prevalencia más bajos en comparación con la clase más alta. Este hallazgo puede reflejar patrones complejos y específicos del contexto de la transición nutricional, en los que el sobrepeso no se distribuye de manera uniforme entre los estratos socioeconómicos. El embarazo de alto riesgo también se asoció con el IMC para la edad, lo que sugiere que las condiciones gestacionales adversas pueden tener implicaciones en las trayectorias de crecimiento temprano^(29,30,31,32,33)^.

Por el contrario, no se observaron asociaciones consistentes entre las variables evaluadas y la estatura para la edad. En general, los hallazgos indican que los resultados antropométricos pueden verse influidos de manera diferente por factores contextuales y de la primera infancia, particularmente en relación con el exceso de peso.

Estos hallazgos deben interpretarse con cautela, ya que los amplios intervalos de confianza observados en varias estimaciones indican una precisión limitada. Además, el diseño transversal no permite establecer relaciones temporales o causales entre las exposiciones y los resultados. La confusión residual, el pequeño tamaño de los subgrupos y la variabilidad en la distribución de la exposición también pueden haber influido en los resultados.

Por último, debido a la naturaleza transversal del estudio, los hallazgos deben considerarse exploratorios y generadores de hipótesis, lo que refuerza la necesidad de estudios longitudinales para dilucidar mejor el papel de las condiciones de la primera infancia y los determinantes sociales en la configuración de las trayectorias de crecimiento infantil.

## Limitaciones del Estudio

Se deben reconocer algunas limitaciones de este estudio. Debido a su diseño transversal, no es posible establecer relaciones temporales o causales entre el estado nutricional y las variables de exposición analizadas. Además, el estudio se llevó a cabo en un solo municipio e incluyó a niños inscritos en guarderías municipales, lo que puede limitar la generalización de los hallazgos a otras poblaciones y contextos.

La no participación debido a la negativa o a datos incompletos puede haber introducido un sesgo de selección, aunque la tasa de participación fue alta (79,8 %). Además, el reducido número de participantes en ciertos subgrupos neonatales, en particular los de nacidos prematuros y con bajo peso al nacer, puede haber reducido la potencia estadística, lo que contribuyó a los amplios intervalos de confianza observados en algunas estimaciones multivariables. Aunque se aplicó la prueba exacta de Fisher cuando fue apropiado, las frecuencias de celdas pequeñas aún pueden limitar la solidez de algunas comparaciones.

La información sobre el historial gestacional y las condiciones socioeconómicas se obtuvo a través de informes de los padres o tutores legales y, por lo tanto, puede estar sujeta a un sesgo de recuerdo. No obstante, el uso de instrumentos estandarizados y validados, junto con la coherencia de los hallazgos con la literatura existente, respalda la validez interna del estudio.

## CONCLUSIÓN

El presente estudio identificó una alta prevalencia de baja estatura y sobrepeso entre los niños inscritos en los Centros de Educación Infantil municipales, con proporciones que superan las reportadas en las encuestas nacionales.

En los análisis ajustados, el índice de masa corporal para la edad se asoció con el peso al nacer, la inseguridad alimentaria, la clasificación socioeconómica y el embarazo de alto riesgo, lo que pone de relieve la influencia tanto de las condiciones de la primera infancia como de los determinantes sociales en el sobrepeso infantil. Por el contrario, no se observaron asociaciones estadísticamente significativas para la estatura para la edad.

La alta prevalencia de la inseguridad alimentaria y nutricional subraya aún más la persistencia de vulnerabilidades sociales y económicas que pueden afectar negativamente la salud y el desarrollo infantil. Estos hallazgos refuerzan la importancia de monitorear continuamente el estado nutricional en la primera infancia y el fortalecimiento de políticas integradas de salud pública que aborden tanto los determinantes biológicos como los sociales, particularmente en contextos socialmente vulnerables.

## Data Availability

El conjunto de datos que respaldan las conclusiones de este estudio está disponible previa solicitud al autor correspondiente.
